# Computerized Tailored Interventions to Enhance Prevention and Screening for Hepatitis C Virus Among People Who Inject Drugs: Protocol for a Randomized Pilot Study

**DOI:** 10.2196/resprot.4830

**Published:** 2016-01-22

**Authors:** Ryan P Westergaard, Shawnika J Hull, Alana Merkow, Laura K Stephens, Karli R Hochstatter, Heidi K Olson-Streed, Lisa M Baker, Timothy M Hess

**Affiliations:** ^1^Division of Infectious DiseasesDepartment of MedicineUniversity of Wisconsin School of Medicine and Public HealthMadison, WIUnited States; ^2^Public Health Communication and Marketing ProgramDepartment of Prevention and Community HealthMilken Institute School of Public Health, The George Washington UniversityWashington, DC, DCUnited States; ^3^Prevention ServicesAIDS Resource Center of WisconsinMilwaukee, WIUnited States

**Keywords:** hepatitis C, substance abuse, intravenous, needle exchange programs, health behavior

## Abstract

**Background:**

Hepatitis C virus (HCV) infection is a growing problem among people who inject drugs. Strategies to reduce disease transmission (eg, syringe exchange programs) and facilitate HCV screening and linkage are available but are under-utilized in many communities affected by injection drug use. Novel approaches to increasing the use of these strategies are needed.

**Objective:**

The goals of this project are to (1) develop and pilot test a computerized tailored intervention for increasing HCV screening and decreasing risky drug use behavior among people who inject drugs and (2) determine the feasibility of disseminating such an intervention using peer-based referrals in the setting of a community-based syringe exchange program.

**Methods:**

This 2-arm, randomized pilot study is being conducted in a large-volume, multisite syringe exchange program in southern Wisconsin. A social network–based strategy was used to recruit a total of 235 adults who reported past-month injection of opioids, cocaine, or methamphetamine. Network recruiters were identified among clients requesting services from the syringe exchange program and were enlisted to refer eligible peers to the study. All participants completed a computer-adapted questionnaire eliciting information about risk behaviors and their knowledge, attitudes, and prior experiences related to HCV screening. Subjects were then randomly assigned to receive usual care, consisting of standard counseling by syringe exchange staff, or the Hep-Net intervention, which provides algorithm-based, real-time tailored feedback and recommendations for behavior change in the style of motivational interviewing. Changes in drug use behaviors and attitudes will be assessed during a second session between 90 and 180 days after the baseline visit. Frequency of repeat HCV testing and HCV incidence will be assessed through a database search 1 year after study completion.

**Results:**

Recruitment for this study was completed in April 2015. Follow-up of enrolled participants is expected to continue until March 2016. Network recruiters were enrolled who referred a total of 195 eligible peers (overall N=235). At baseline, the median age was 34 years; 41.3% (97/235) were non-white; and 86.4% (203/235) reported predominantly injecting heroin. Most participants (161/234, 68.8%) reported sharing injection equipment in the past and of these, 30.4% (49/161) had never been tested for HCV.

**Conclusions:**

This study will provide preliminary evidence to determine whether incorporating computerized behavioral interventions into existing prevention services at syringe exchange programs can lead to adoption of healthier behaviors.

**Trial Registration:**

ClinicalTrials.gov NCT02474043; https://clinicaltrials.gov/ct2/show/NCT02474043 (Archived by WebCite at http://www.webcitation.org/6dbjUQG7J)

## Introduction

### Objectives

The overall goal of this project is to explore whether deploying a computer-adapted behavioral intervention coupled with onsite, rapid hepatitis C virus (HCV) screening is a feasible and acceptable approach to reducing transmission risk behavior and improving HCV case detection in the setting of a syringe exchange program. The intervention described in this paper incorporates lessons from formative research conducted with the target population [1] and prior experience implementing social network strategies for HIV testing within community-based prevention agencies [2]. In this manuscript, we describe the development of the Hep-Net intervention and its implementation and evaluation through a pilot randomized controlled trial (RCT). We present baseline data describing the participants enrolled and discuss challenges encountered disseminating the intervention using peer-based referrals.

### Background

Epidemiologic studies suggest that HCV transmission is increasingly driven by injection drug use among young adults in rural and suburban settings. A cluster investigation in 6 contiguous rural counties in northern Wisconsin found that the number of HCV infections reported annually increased by more than 200% between the periods 2004-2008 and 2009-2010 [3]. Among individuals newly diagnosed with HCV in this outbreak, 94% reported a history of sharing needles or other drug preparation equipment. In this investigation and in similar outbreaks in Massachusetts [4], rural Indiana [5], and several Appalachian states [6], many young adults described a history of injecting prescription opioid medications for several years before transitioning to injecting heroin or methamphetamine. These sharp increases in HCV incidence concentrated in communities with traditionally poor access to prevention services highlight the need for evidence-based, targeted interventions to reduce HCV transmission and coordinate efforts to increase HCV testing and linkage to treatment for those who are infected [7].

Syringe exchange programs are a widely used strategy to reduce harm related to injection drug use. Numerous observational studies support the effectiveness of syringe programs for reducing behaviors leading to transmission of HIV and viral hepatitis and increasing entry into drug treatment programs [8-11]. Ensuring the availability of sterile syringes and other drug injection equipment, while a necessary component of disease prevention for people who inject drugs, is only one of several strategies that can be implemented through syringe exchange programs [12,13]. Other important components of risk reduction include linkage to addiction treatment, overdose prevention, and testing and linkage to care for HIV, viral hepatitis, and sexually transmitted infections. However, resource limitations pose challenges to consistently delivering multicomponent services that meet the diverse needs of people who inject drugs. Syringe exchange programs face resource limitations that are driven by social and political factors such as prohibitions on federal funding and local opposition. Many syringe exchange programs have insufficient resources to provide adequate syringe coverage or deliver a full package of preventative services to their clients [14]. Further, even when prevention services are available in the community and are of no cost to clients, many high-risk individuals still cannot or do not access syringe exchange programs due to myriad environmental and psychosocial barriers. As a result, many people who inject drugs are not regularly engaged in prevention services [15].

### Novel Approaches

#### Computerized Interventions

Computer-based interventions deployed in syringe exchange programs or other community-based settings may represent a promising, low-cost strategy for delivering tailored health information that is specific to the needs of people who inject drugs. Studies examining computer-tailored interventions (CTIs) have shown positive behavior changes in a wide range of contexts, including alcohol reduction in college students, preconception care in women, and HIV prevention among juvenile offenders and drug users [16-19]. A meta-analysis of 88 CTIs showed a significant effect size for behavior change in smoking cessation, mammography, physical activity, and dietary practices, indicating CTIs have a clinically significant impact on rates of behavioral risk factors [20].

CTIs assess individual behavior, environmental barriers, and psychosocial determinants of positive behavior change. They then use data-driven decision guidelines to construct automatic, tailored feedback providing each individual with a personalized approach to risk reduction. CTIs are mobile, user-friendly, and brief. As such, CTIs may have an advantage in engaging transient, hard-to-reach populations in resource-constrained prevention settings such as syringe exchange programs.

#### Extending Prevention Services Through Social Networks

Strategies to increase engagement in prevention services must address environmental barriers such as geographic inaccessibility and psychosocial barriers related to individual motivation and behavioral skills. Social network-based strategies, which have been developed and implemented in many US cities to increase HIV testing, may facilitate dissemination of prevention services through both of these domains. In a demonstration project funded by the Centers for Disease Control and Prevention (US Department of Health and Human Services) conducted in 7 US cities, 5.6% of clients recruited through peer referrals were HIV positive compared to a prevalence of approximately 1% who self-referred [21-23]. Programs to promote HIV testing have taken advantage of existing social networks and the meaningful influence of peers by enlisting high-risk clients to recruit, refer, or otherwise encourage their associates to participate in testing. Adaptation of this strategy to deliver HCV testing and prevention services to people who inject drugs was one of the factors motivating the development of this project.

## Methods

### Study Design

#### The Hep-Net Intervention: Overall Objectives

This project has two main objectives. First, it aims to determine whether a CTI is a feasible and acceptable approach to increasing readiness to engage in various health-promoting behaviors among people who inject drugs. It targets 4 different behavioral domains: (1) undergoing regular HCV screening, (2) using clean works for every injection, (3) taking steps to prevent opioid overdose, and (4) reducing and ultimately ceasing injection drug use. The second objective is to determine the feasibility of using social networks to expand delivery of computerized prevention interventions to hard-to-reach people who inject drugs.

#### Theoretical Frameworks

**Figure 1:**
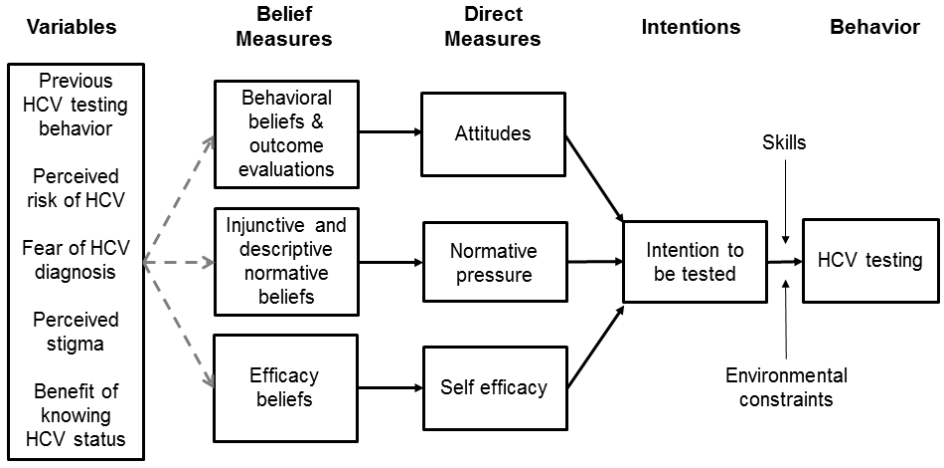
The Integrative Model (adapted from Fishbein [24]).

Hep-Net is grounded in behavior change theory and motivational interviewing techniques. The guiding behavioral theory is the integrative model of behavior change, which is schematized in Figure 1 [24,25]. The integrative model and its historical predecessors, the theory of planned behavior [26] and the theory of reasoned action [27], are supported by evidence across a wide array of health behaviors and populations [28-31]. The integrative model expands the scope of the theory of planned behavior and the theory of reasoned action by acknowledging the importance of skills, abilities, and environmental constraints as moderators of the relationship between behavioral intention and action [32].

Hep-Net is also guided by the transtheoretical model (ie, stages of change), which assumes that behavior change should be considered a continuum rather than a dichotomy [33,34]. Specifically, behavior change occurs through a series of stages in which individuals can move back and forth. In the precontemplation stage, individuals are not yet considering behavior change. In the contemplation stage, individuals may be considering change but have not yet taken steps toward behavioral change. Contemplation is followed by preparation, action, and maintenance. Although the stages are considered serial, one may skip particular stages (eg, planning/preparation) and, at any point in the continuum, one may digress to a previous stage of readiness (ie, from action to preparation). The Hep-Net system uses motivational interviewing techniques, which are founded on the transtheoretical model, to assess readiness for change with respect to safe injection practices, substance use reduction, and overdose prevention. The model also informed the types of specific feedback and risk reduction activities suggested to participants, as described below in the discussion of the risk reduction exercise.

#### Formative Research and Intervention Development

Using the integrative and transtheoretical models as the guiding frameworks, the system assesses participants’ risks and protective behaviors; preferences for behavior change domain (eg, decreasing risky injection practices, reducing opioid use, overdose prevention); and attitudinal, normative, and efficacy beliefs. The system then tailors the content of the intervention to the individuals’ stage of readiness for change, salient beliefs, and the chosen behavioral target (eg, safer injection practices). To develop appropriate questionnaire items and health-promotion messages that address relevant beliefs held by the target population, we analyzed formative data collected through an anonymous cross-sectional survey of 553 syringe exchange clients [1]. Health promotion messages specifically tailored to individual stages of readiness to change were developed collaboratively by a research team of investigators with expertise in health communications, counseling psychology, and clinical medicine. Draft messages were discussed at in-person meetings and later pilot tested with several end-users who were current or previous clients of the syringe exchange program to elicit feedback to inform further refinement of messages.

The risk assessment survey and tailored behavioral computerized intervention were delivered using an Internet-based, customizable, interactive software tool developed in consultation with DatStat Inc (Seattle, WA). DatStat performed all necessary programming for creation of the intervention. The tool provided a patient-driven counseling experience similar to those used in other studies of high-risk populations [35,36]. The intervention simulated a motivational interview, asking contextually appropriate questions, and was intended to capture the essence of a patient clinical experience on a computer. In real time, the program synthesized patient responses about risks, knowledge, and beliefs; presented a list of risk factors to the participant based on those responses; and then guided the participant in developing an individualized risk reduction plan.

After developing and pilot testing the intervention with a small number of volunteers, we proceeded to recruitment and enrollment in the pilot RCT. The study protocol was reviewed and approved as a minimal risk study by the Health Sciences Institutional Review Board at the University of Wisconsin-Madison.

#### Study Population, Recruitment, Eligibility, and Screening

Enrollment in the study began in September 2014, and all baseline assessments were completed by April 2015. Participants in the study were either clients of an established, multisite syringe exchange program operating in southern Wisconsin or peers recruited from the social networks of these clients. Eligibility criteria included age 18 years or older, injection drug use in the past 30 days, and willingness to provide contact information for the 3-month follow-up. Pregnant women and people who did not speak English were excluded. As one goal of the study is to conduct outreach among high-risk populations who do not regularly use prevention services, we used social network–based referrals to recruit the majority of the study sample. Syringe exchange clients were informed about the study and screened for eligibility during a routine encounter at the syringe exchange program. Upon completion of the baseline visit, study participants received referral coupons and were encouraged to refer eligible peers. Coupons were marked with a unique code number used to track referral chains. Participants received US $10 in cash as compensation for time spent completing the baseline study encounter and an additional US $10 for each eligible peer they referred (up to 5) who enrolled in the study.

#### Baseline Study Assessment

At the initial study encounter, participants were encouraged to get a rapid HCV antibody test unless they had had a positive HCV antibody test in the past or had gotten a rapid HCV test within the last 3 months. Receiving an HCV test was not a requirement for participation in the study; it is a standard service offered to all syringe exchange clients. The computerized survey was designed to last 20 to 30 minutes. For those consenting to HCV testing, the baseline assessment was administered after participants provided a fingerstick blood specimen for the rapid HCV test, allowing them time to complete most of the baseline assessment while awaiting the test results. The complete baseline questionnaire is reproduced in Multimedia Appendix 1.

Question items were developed based on the integrative model to evaluate attitudes, norms, and self-efficacy beliefs relevant to each of the targeted health behaviors. Table 1 displays sample question items assessing the relevant constructs in the integrative model for the behavior of HCV testing. Response options to each of these questions were a 5-point Likert scale ranging from “strongly disagree” to “strongly agree.” Participants rated their readiness to make changes toward each of 4 behavioral goals: (1) “I will cut down on my drug use or quit using drugs completely,” (2) “I will use clean needles, cottons, and cookers every time I inject drugs,” (3) “I will get tested for hepatitis C every 6 months for as long as I’m using,” and (4) “I will get trained to give naloxone (or Narcan) in case someone I am with has an overdose.”

**Table 1:** Examples of questions based on the integrative model.

Survey question	Integrated model domain
“I am confident that if I really wanted to, I could get tested for hepatitis C every six months, for as long as I am shooting drugs.”	Self-efficacy
“Most people who are important to me think I should get tested for hepatitis C.”	Injunctive normative beliefs
“Most people who are similar to me have been tested for hepatitis C.”	Descriptive normative beliefs
“Getting tested for hepatitis C is important to me.”	Attitudes

**Figure 2:**
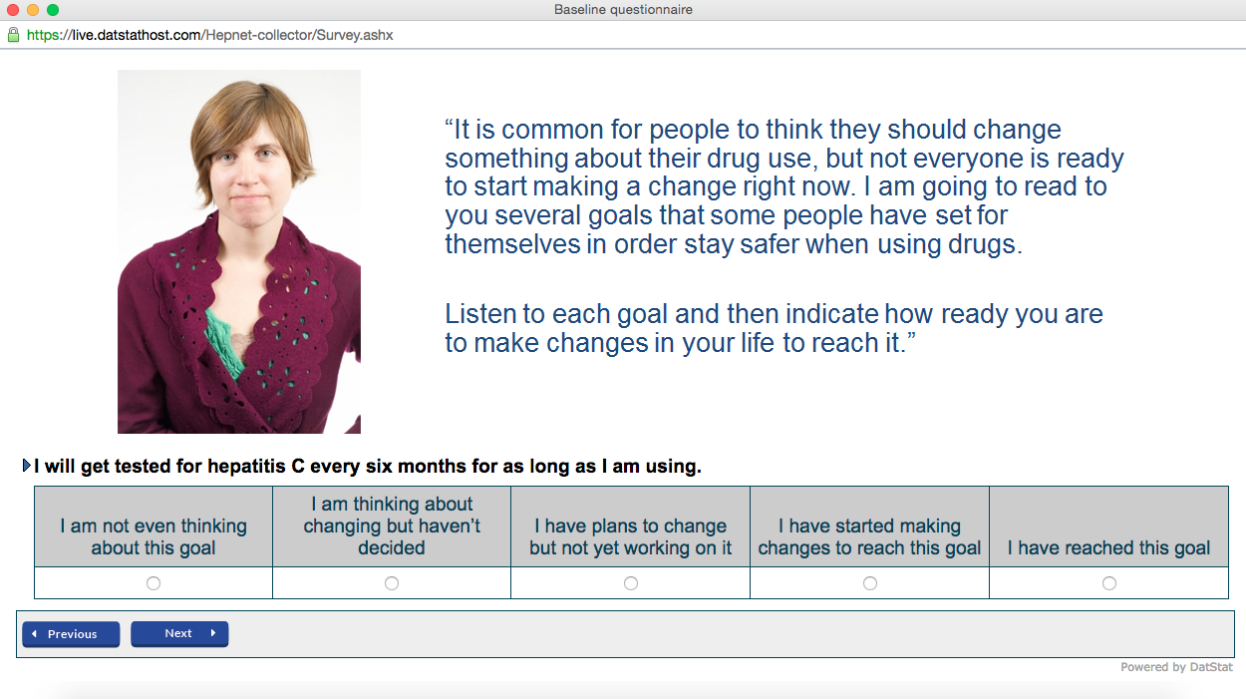
Assessment of readiness to change based on the Transtheoretical Model.

Using the transtheoretical model, we assessed readiness to adopt specific healthy behaviors using the visual analog scale shown in Figure 2. Each behavior was characterized as a health-related goal that a person who injects drugs may have, and respondents were asked to characterize their readiness to adopt the behavior by selecting a statement on the spectrum of “I am not even thinking about this goal” (precontemplation stage) to “I have reached this goal” (maintenance stage). For the intervention group, the stage of change reported by respondents during the baseline session was used in the algorithms to determine what tailored content would be displayed in the subsequent risk reduction intervention.

The baseline questionnaire included basic demographic and locator information, including multiple means of electronic communication (eg, text messaging, email, Facebook) to facilitate coordination of follow-up. Additional sections included a risk behavior questionnaire assessing addiction severity, overdose risk, and injection-related and sexual behaviors associated with transmission of HCV. To maximize accurate disclosure of high-risk and sensitive behaviors, both the baseline questionnaire and the tailored intervention used audio computer-assisted self-interview. As an enhancement designed to better simulate a motivational interviewing session, photographs of a model portraying a counselor were embedded in the survey program to accompany the audio-recorded instructions, survey questions, and, if applicable, tailored feedback messages. At the beginning of the session, participants selected 1 of 3 female avatars that appeared to have varying racial/ethnic backgrounds. The avatar selected by the participant would be displayed throughout the baseline session and the subsequent follow-up assessment 3 months later.

#### Randomization

After completion of all required sections of the baseline survey, participants were randomly assigned to receive the risk reduction intervention or be in the control group, in which the computer session terminated after completion of the survey. Both the intervention and control groups received individualized prevention counseling per standard of care at the syringe exchange programs. Challenges related to data synchronization across multiple sites and the need for offline data collection in rural communities made stratified, block randomization infeasible. Therefore, simple randomization was used to place participants to either group using the survey software during the baseline session.

### Intervention Content

#### Overview

Participants assigned to receive the risk reduction intervention were presented with a series of screens featuring text and audio content summarizing the participant’s risk behaviors and delivering health-promotion messages. Components of the tailored intervention consisted of an overall risk synthesis, selection of behavioral goal, and individualized risk reduction exercise and assessment of self-efficacy related to risk reduction plan. An example of the series of content screens displayed for a participant in the precontemplation stage with respect to overdose prevention is reproduced in Multimedia Appendix 2.

#### Risk Synthesis

The risk synthesis was introduced with 1 screen displaying positively framed feedback messages emphasizing behaviors reported by the participant that can reduce risk of HCV transmission and/or opioid overdose. A subsequent screen displayed tailored feedback regarding specific behaviors associated with increased risk reported by the participant.

#### Selection of Behavioral Goal

In the second portion of the intervention, participants chose a behavioral goal they would like to work on over the next 3 months (HCV testing, Narcan training, use of clean needles or works, or reducing or suspending drug use) and selected action steps tailored to the participants’ assessed stage of change within that one risk category. The relevant stage of change was determined by responses to questions addressing the risk reduction goals.

After selecting a behavioral risk reduction goal, participants received a message introducing the activity that was tailored to their self-reported stage of readiness to change, relevant to that particular risk category. Participants were then guided through a series of screens which gave feedback and educational content (eg, the opportunity to view a brief video) tailored to the types of risk behaviors and stage of readiness to change. Messages were designed to encourage movement along the stages of change in the direction of action/maintenance.

#### Individualized Risk Reduction Exercise

The final intervention component was an interactive activity in which participants were asked to create a risk reduction plan. Participants were presented with a list of 10 to 12 possible suggested action steps related to the risk reduction goal they selected. The content of these lists was informed by the transtheoretical model, encouraging incremental change through concrete actions that could be taken. Participants were asked to select 3 to 5 of the steps they felt they would be able to do in the ensuing 3 months. After participants selected the steps, their individualized risk reduction plan was read back to them. They were asked to confirm their selected steps and given an opportunity to go back and change their choices. Finally, participants were asked to answer a series of questions assessing confidence in their ability to complete their plan.

#### Follow-Up Assessments and Outcome Measures

Upon completion of the baseline session, participants were reminded of the need to return for a follow-up assessment 3 months after the baseline session. The second study visit uses an Internet-based survey designed to assess the same behavioral and attitudinal domains captured through the baseline questionnaire. This allows us to evaluate any temporal changes in the frequency of substance use, injection risk behaviors, overdose risk, and HCV testing. The follow-up assessment also evaluates self-reported readiness to change, attitudes, and perceived norms, providing an opportunity to detect whether the tailored intervention may influence outcomes through these intermediate variables.

In addition to repeating the assessment of baseline variables for longitudinal analysis, the follow-up questionnaire captures information on participant perceptions about whether they met any of the health goals discussed in the baseline session. For participants determined to be HCV-infected at baseline, the survey assesses whether they received any follow-up testing or medical care for HCV since receiving their test result. Finally, it evaluates usability and acceptability of the intervention through a series of questions delivered to participants who were randomized to the active study arm. The additional questionnaire items used during the follow-up assessment are presented in Multimedia Appendix 3.

There are multiple behavioral outcomes of interest in this project. Accurate assessment of drug use behaviors may be limited because it relies on participant self-report and may be biased due to losses to follow-up. HCV testing behavior may be captured with greater validity because it will be ascertained by searching HCV testing data collected and reported by all agencies receiving funding from the Wisconsin Division of Public Health. For participants who have received a reactive HCV screening test result, we will determine whether any follow-up testing was performed, including confirmatory HCV RNA or HCV genotype tests, which would indicated that the participant was linked to evaluation and/or treatment of HCV.

#### Sample Size and Power

Demonstrating efficacy of the intervention through detection of a statistically significant effect size was not a primary goal of the project. However, we considered it plausible that the intervention might significantly influence participants’ decisions to receive HCV testing even after a single session. While planning the pilot trial, we calculated a target sample size that would be sufficient to detect what we considered to be a meaningful difference in the proportion of participants who undergo HCV testing within 12 months after enrollment. Assuming that approximately 10% of participants would be known to have HCV infection at enrollment and accounting for expected losses to follow-up, we estimated that a sample size of 408 would provide 90% power to detect a difference of 0.15 in the proportion of participants who voluntarily returned for an HCV test within a year of enrollment.

We believed that achieving a target sample size of 408 was feasible and justified based on data from the pilot survey conducted in 2012 reporting that 69.4% of syringe exchange clients had an HCV test in the prior 12 months and 14.9% of clients had ever had a positive HCV test [1]. Because this intervention was targeting people who inject drugs but may not be regular users of the syringe exchange program, we anticipated that the number reporting prior HCV testing would be lower. We believed an effect size of this magnitude was reasonable based on prior meta-analyses of tailored communication interventions, which provide support for moderate mean intervention effect sizes [20] across a variety of health behaviors, including addiction-related behaviors (eg, smoking) and HIV risk-related behaviors [37]. Notably, findings suggested effect sizes increased with the number of behaviors intervened upon, with mean effect size of g=.24 (95% CI 0.18-0.31) for interventions focused on 3 behaviors [20].

#### Data Analysis

Descriptive statistics reported here were calculated for baseline variables using SAS 9.3 (SAS Institute Inc). Differences in participant characteristics were assessed using a Wilcoxon rank-based general linear model approach for continuous variables and Pearson chi-square or Fisher exact tests for differences in categorical variables.

To evaluate the effectiveness of the risk reduction intervention, we will test within-group changes from baseline to follow-up (eg, change in frequency of needle-sharing) using the matched-pair Wilcoxon rank-sum test. Similarly, binary outcome changes will be examined using either the McNemar test or Fisher exact test.

To assess the independent role of the intervention in improving screening for HCV, we will use a logistic regression model on the binary outcome of having an HCV test within the 12-month period postintervention. Again, a Wilcoxon general linear model framework will be used for analysis of Likert scale outcome measures. Indicators pertaining to individual level outcomes within intervention and control groups will be measured among the same individuals at baseline and follow-up.

To avoid potential sampling bias toward individuals with larger networks, we will adjust regression models using inverse probability weights based on individual recruitment network sizes [33]. To account for correlation between recruiter and recruited, we will create a variable indicating who the recruiter of each subject was and use it as a cluster variable using generalized estimating equations. An exchangeable correlation structure within each cluster will be assumed (ie, correlation between any 2 subjects recruited by the same recruiter will be assumed to be the same). For all tests, a 2-sided *P* value of less than .05 is considered statistically significant.

## Results

Between September 2014 and April 2015, 235 people completed the baseline survey. Baseline descriptive characteristics of the sample are displayed in Table 2. The racial/ethnic breakdown was reflective of the general population of the region, with non-Hispanic whites comprising 60.3% (129/232) of the sample and those self-described as black or African American comprising 28.1% (66/235). The median age was 35 years (range 18-63 years). While most participants who provided a valid address (131/219, 59.8%) lived in the city of Milwaukee, about one-third (70/219, 29.0%) of participants resided in a municipality with a population less than 50,000 residents, including 17.8% (39/219) who lived in a city with a population less than 5,000. Comparison of the control and intervention groups with respect to baseline characteristics demonstrated no statistically significant differences, assuming a 2-sided alpha of .05.

**Table 2:** Baseline characteristics by intervention group (N=235).

Characteristics	Category	Control (N=126)	Intervention (N=109)
Age, years, median (IQR)		35 (28-46)	33 (27-44)
Gender, n (%)	Male	92 (73.0)	89 (81.7)
	Female	34 (27.0)	20 (18.3)
Race, n (%)	White	79 (63.0)	59 (54.1)
	Black	32 (25.0)	34 (31.2)
	Other or multiple	15 (12.0)	16 (14.7)
Ethnicity, n (%)	Non-Hispanic/Latino	112 (90.3)	102 (94.4)
	Hispanic/Latino	12 (9.7)	6 (5.6)
Highest education level, n (%)	Less than high school	17 (13.5)	7 (6.4)
	HS diploma or GED	61 (48.4)	51 (46.8)
	Some college or vocational school	45 (35.7)	47 (43.1)
	College degree	3 (2.4)	4 (3.7)
Currently employed, n (%)	No	92 (73.0)	69 (64.0)
	Yes	34 (27.0)	39 (36.0)
Legal income in last year, n (%)	None	31 (26.0)	30 (27.5)
	US $1-11,500	62 (51.0)	49 (45.0)
	More than US $11,500	28 (23.0)	30 (27.5)
Homeless during the past year, n (%)	No	58 (46.0)	52 (48.0)
	Yes	68 (54.0)	56 (52.0)
Incarcerated during the past year, n (%)	No	82 (66.0)	63 (59.0)
	Yes	42 (34.0)	44 (41.0)
Has health insurance, n (%)	No	20 (16.0)	11 (10.0)
	Yes	105 (84.0)	98 (90.0)
Has primary care provider, n (%)	No	53 (42.4)	39 (36.0)
	Yes	72 (57.6)	69 (64.0)

Of the 235 participants who completed the baseline assessment, 80 (34.0%) agreed to receive a rapid HCV test, and 14 (17.5%) tests were reactive. The most common reasons given for declining the test were the participant had been previously tested (36/155, 23.2%), did not want to or did not feel ready to be tested (22/155, 14.2%), already knew he or she was HCV-positive (17/155, 11.0%), and did not have enough time (13/155, 8.0%).

Drug use characteristics of the baseline sample are displayed in Table 3. Heroin was the drug most frequently injected by participants and nearly half (105/235, 44.7%) reported that they inject on a daily basis. Most participants (161/234, 68.8%) reported they had shared syringes, cotton filters, or cookers with other people while injecting drugs in the past. Of those reporting sharing injection equipment in the past, 44.1% (71/161) had shared syringes, cottons, or cookers during the past 3 months, and 30.4% (49/161) reported they had never been tested for HCV.

**Table 3:** Drug use characteristics by intervention group (N=235).

Characteristics	Category	Control (N=126) n (%)	Intervention (N=109) n (%)
Drugs injected in past 30 days	Heroin	111 (88.1)	92 (84.4)
	Prescription opioids	26 (20.6)	21 (19.3)
	Cocaine	36 (28.6)	35 (32.1)
	Methamphetamine	1 (0.8)	6 (5.5)
Frequency of drug injecting in past 30 days	Less than daily	67 (53.2)	63 (57.8)
	Every day	59 (46.8)	46 (42.2)
Has shared needles, cottons, or cookers	No	37 (29.6)	36 (33.0)
	Yes	88 (70.4)	73 (67.0)
Has had an opioid overdose	No	75 (60.5)	70 (64.2)
	Yes	49 (39.5)	39 (35.8)

**Figure 3:**
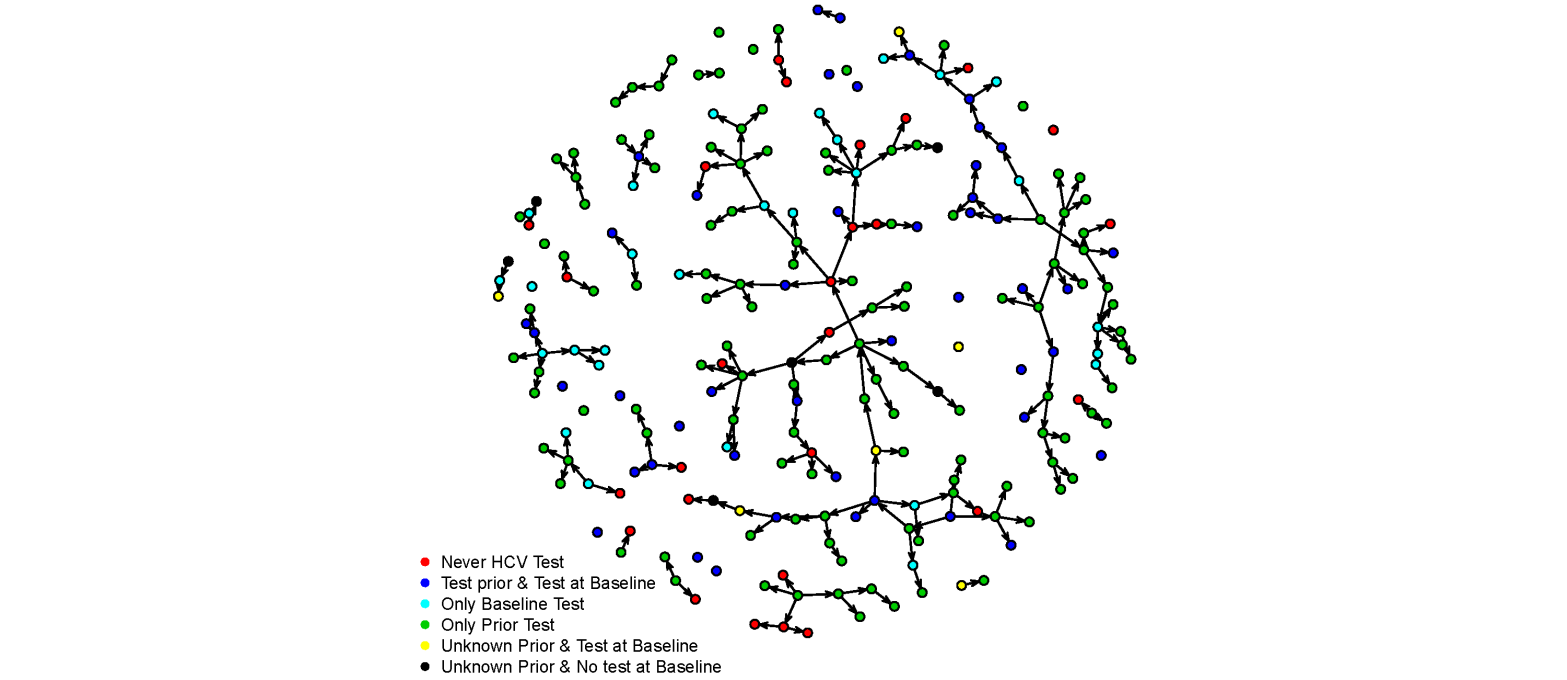
Network diagram of peer referral chains by hepatitis C virus testing history.

Using social networks to recruit participants allowed us to reach a population that may have otherwise not been reached. In the first phase of the study, prevention staff recruited 40 individuals to participate who were existing clients of the syringe exchange program. These participants referred 195 peers who were determined to be eligible and were enrolled in the study. As shown in Figure 3, linking participants via referral chains allows visualization of 2 large networks and several smaller ones.

## Discussion

### Main Findings

The overarching goal of this pilot RCT was to determine if a computerized, tailored intervention was feasible and acceptable to implement in an syringe exchange program. Though its potential impact in increasing HCV testing, overdose prevention, and use of clean syringes and works will be reviewed after all follow-up assessments and HCV testing follow-up data are collected, the data collected to date demonstrate that the approach is feasible overall. There was a high level of willingness to participate, and many participants referred peers, indicating acceptability of this type of intervention.

### Unanticipated Challenges

Throughout the initial period of enrollment and completion of baseline assessments, the study staff encountered several unanticipated challenges. Scheduling appointments for enrollment and completion of study assessments proved to be difficult; participants preferred to drop in at a time that was convenient for them. Often, individuals forgot about their appointments, did not have transportation to make it to their scheduled appointment, and could not be reminded via phone or text because they did not own a cell phone or have minutes. Both study sites amended their procedures and became accessible for drop-in appointments to accommodate the difficulties faced by people who inject drugs in keeping scheduled appointments.

The Hep-Net study was designed as a pragmatic intervention trial rather than a highly controlled clinical trial, which would have required more resources and been less generalizable to other settings. Prevention staff had numerous responsibilities that superseded the tasks they were asked to complete for the research project, such as obtaining informed consent and administering the computerized survey. Balancing these competing demands required communication and an effective partnership among the research team, the existing prevention staff, and administrators of the syringe exchange program. Within 2 months of enrolling the first participants, study site staff began to identify the best time periods for survey visits and how to best fit the intervention into routine prevention services. When the study team and prevention staff determined how to best achieve balance between research and service activities, it became obvious that the most appropriate rate of accruing new subjects would not allow recruitment of a sample as large as originally planned. The target sample size was modified after several months for this reason to the more realistic goal of 120 subjects per group.

Computer literacy was another unanticipated challenge experienced by study staff. Many participants had little background using computers and struggled to understand how to answer questions and advance the program. Although site staff made themselves available for computer questions and aided participants in computer fluidity, several participants took over an hour to complete the survey rather than the anticipated half hour.

Finally, Internet connectivity and a private space to screen and deliver the survey were difficult barriers to overcome. Neither syringe exchange program had readily available WiFi, so Ethernet accessibility was imperative in collecting and synchronizing survey data. Private spaces with Ethernet accessibility were difficult to keep consistently vacant of other prevention services, which made data collection more difficult. Furthermore, a substantial number of study assessments were completed via mobile syringe exchange outreach where no Internet connectivity was available. The remote data collection feature in the DatStat software package was used for these surveys, which added a layer of complexity causing some frustration among participants and staff.

The main limitation of this study is its lack of generalizability to other cities and states and other local epidemics. While Hep-Net may be feasibly used in the context of Wisconsin’s opioid epidemic, it may not apply to other geographical areas targeting the same population. This CTI used existing preliminary data from a pilot study of over 500 Wisconsinites to tailor the intervention content to a specific population. Additionally, our sample was subject to selection bias because individuals using a syringe exchange program tend to be a healthier, higher-functioning subset of people who inject drugs. Although the computerized approach to data collection was designed for complete anonymity to reduce social desirability bias, participants may have answered questions to please researchers.

A major strength of this study is the use of well-established syringe exchange programs as home to Hep-Net. The community-based prevention specialists who implemented this project are highly regarded among community members and have built trust over years of service. Without their involvement, acceptability of the intervention would likely have been much lower.

### Conclusion

If effective, Hep-Net has the possibility to facilitate a more comprehensive approach to prevention and linkage to care within syringe exchanges and other community-based programs. Syringe exchange programs, while shown to be effective, are already understaffed and lack resources. Hep-Net’s role is to fill in the gaps presented by agency challenges to provide behavioral care to a subset of a community that is substantially underserved. To disseminate the intervention, busy syringe exchange programs could present Hep-Net to clients or use the Internet to reach rural or immobile populations. We hope the results will lead to implementation of a CTI in community-based settings. If the study hypotheses are confirmed, the proposed solution can be tailored to specific cities and states and disseminated to reduce the impact of hepatitis C among people who inject drugs.

## Multimedia Appendix 1

Baseline questionnaire.

## Multimedia Appendix 2

Sample content screens from Hep-Net intervention.

## Multimedia Appendix 3

Follow-up assessment.

## Abbreviations

CTI: computer-tailored interventions
HCV: hepatitis C virus
RCT: randomized controlled trial
