# Supplementary material for: Computerized Tailored Interventions to Enhance Prevention and Screening for Hepatitis C Virus Among People Who Inject Drugs: Protocol for a Randomized Pilot Study
Source: JMIR Res Protoc. 2016 Jan 22;5(1):e15. doi: 10.2196/resprot.4830 (PMC4744331; doi:10.2196/resprot.4830)
Supplement: Supplementary file 1 [file resprot_v5i1e15_app1.pdf]

Appendix 1. Baseline Assessment [Interviewer-administered]

Oral consent script
This research study involves using a computer survey to learn about people's risk of hepatitis C and other problems related to injecting drugs. To decide whether you are eligible for the study, I would like to ask you a few short screening questions. You do not have to answer them if you don't want to. Your answers to these questions will be kept confidential. If you don't qualify for the study or choose not to participate, we will not keep any information about you that could be used to identify you.

Enter the number on the referral coupon
___ ___ ___ ___ - ___ ___

How old are you?
Enter number _____ (Range 11 – 99)
(If not 18 or older, continue with the following questions but inform the potential participant at the end of the screen that he or she is not eligible to participate.)

What is your gender?
(Not an eligibility requirement)
Male=0
Female=1
Transgender=2

What county do you currently live in?
Enter county name:____________________
(Not an eligibility requirement)

Have you ever been tested for hepatitis C in the past?
0=No
1=Yes
9=Not sure

When was the last time you were tested for HCV?
Enter date of last test: _____/_____/_____
(8 digit date, valid range 01/01/1900 to present date)

(if HCVPOS=yes)
What was the result of your most recent HCV test?
0=No
1=Yes
9=Not sure

Are you currently pregnant?
Yes _____ No _____ I don't know _____
(If pregnant, then **STOP** and inform the subject that she is not eligible for the study, but will be offered conventional HCV antibody testing and referrals for prenatal care and substance abuse treatment)

When was the last time you injected the following?

Cocaine<br>Heroin<br>Methamphetamine (Crystal, Meth, Speed)<br>Prescription pain killers	Never _____<br>More than 1y ago ____<br>Past year _____<br>Past month _____

(If does not report any injecting in the past month, then continue with the following questions but inform the potential participant at the end of the screen that he or she is not eligible to participate.)

Are you willing to give us contact information like your phone number or email address so that we can contact you to complete the second half of this study 3 months from now?
Yes _____ No _____

Eligible for study? [18 or older, injected in past month, not pregnant, willing to give locator info]
Yes _____ No _____

**If the client is eligible, then proceed with informed consent process and HCV testing, as appropriate.

Agreed to HCV test today?
Yes _____ No _____

If declined to participate, enter reason for refusal
Enter text:_________

Participant identification & locator information [Interviewer-administered]

[If agrees to HCV test and enrolls in study, complete following information then proceed with rapid HCV and/or HIV test. The researcher will type this information into the computer.]

Enter Participant ID: ___ ___ ___ ___

Please tell me your full legal name:

____

First Middle Last

Other names, nicknames, or aliases: ______________________________________

Date of Birth: _____ / _____/ _____

Mother's maiden name:____

Last Name

Cell phone number:____

Home/ other phone number:____

Your E-mail address:____

Alternate E-mail addresses:____

Are you on Facebook?: □ Yes □ No

If yes, what email address do you use?:_________________________________

Home Address:____

(Street address) (Apt # or P.O. Box #)

____

(City) (State) (Zip)

Person to contact if we need help contacting you

Name: ____

First Middle Last

Contact person's home phone:____

Contact person's cell phone: ____

How do you prefer to be reminded about your 3 month follow-up visit? (check all that apply)

email ___ text message ___ telephone call ___ other (specify): _____

[After obtaining verbal consent, collect specimen for HCV test, and while awaiting result, click button to “START SURVEY”]

Baseline questionnaire [Self-administered, ACASI]

{Narrated text} Welcome to the Hep-Net Wisconsin project. And thanks for agreeing to participate today. For the next 20 to 30 minutes we are going to find out more about your drug use. We'll review the good things you have been doing to keep yourself safe, and talk about some things you could do differently to avoid health problems in the future.

{Narrated text} First, select one of the pictures below to choose a virtual counselor who will guide you through today's program.

{Narrated text} Hi there! I will be helping you work through the program today. In the first section, we are going to ask you to complete a survey that asks some personal questions about you, your drug use, and health problems you may have had in the past. This should take about 10 to 15 minutes. Everything you say will be kept confidential, so please be as honest as possible.

To begin, let's take a look at some of the different kinds of questions you will be asked to answer during today's program. If you have trouble using the computer or any questions at all, you can pause to ask for assistance from the staff member who is working with you today.

Demographics & training questions

{Narrated text} Some questions ask you to pick a single answer. For example: Do you consider yourself Hispanic or a Latino/Latina?

{Displayed text} Some questions ask you to pick a single answer.

Do you consider yourself Hispanic or a Latino/Latina?
0 = No
1 = Yes

{Narrated text} Thanks. Some questions will ask you to enter a number using the keypad. For example: What was the highest grade you completed in school? If you graduated from high school, enter the number 12.

{Displayed text}: Some questions will ask you to enter a number using the keypad.

What was the highest grade you completed in school? If you graduated from high school, enter the number 12.
Enter number: _____

{Narrated text} Some questions will ask you to choose all answers that apply. For example: Have you attended any of the following other types of schools?

{Displayed text}
Have you attended any of the following other types of schools?
0 = GED or HSED program
1 = Vocational or technical school
2 = College or university
3 = Graduate school

Which categories best describe your racial background? You can choose more than one if necessary.
0 = White
1 = Black or African American
2 = American Indian or Native American
3 = Asian
4 = Hawaiian or Pacific Islander
5 = Other Racial Background

{narrated text} Finally, some questions will ask you to pick a point on a line that shows how strongly you agree or disagree with a statement. We may also ask you to pick a number on a scale of 0 to 100 that indicates a percentage of something. Click on a point right above the line to mark your answer. For example:

Thinking about people living in Wisconsin who also inject drugs, what is your <u>best guess</u> about the percentage who have hepatitis C?

{Displayed text} Some questions will ask you to pick a point on a line that shows how strongly you agree or disagree with a statement

Thinking about people living in Wisconsin who also inject drugs, what is your <u>best guess</u> about the percentage who have hepatitis C?

0%	25%	50%	75%	100%

Stages of Change

Transition slide (Avatar & instructional text only)
{narrated text} Good job. Now we'll move on to the main part of the survey. The main goal of this program is to help people who inject drugs think about ways they can keep themselves safe and healthy. When you're using, it can be hard to completely avoid putting your health at risk.

{Displayed text} Goals:
Stay safe
Stay healthy

{narrated text} It is common for people to think they should change something about their drug use, but not everyone is ready to start making a change right now. I am going to read to you several goals that some people have set for themselves in order stay safer when using drugs. Listen to each goal and then indicate how ready you are to make changes in your life to reach it.

{Displayed text} Listen to each goal and then indicate how ready you are to make changes in your life to reach it.

I will use clean needles, cottons, and cookers every time I inject drugs

I am not even thinking about this goal	I am thinking about changing but haven't decided	I have plans to change but I'm not working on it yet	I have started making changes to reach this goal	I have already reached this goal

I will cut down on my drug use or quit using drugs completely

I am not even thinking about this goal	I am thinking about changing but haven't decided	I have plans to change but not yet working on it	I have started making changes to reach this goal	I have already reached this goal

I will get tested for hepatits C every six months for as long as I am using

I am not even thinking about this goal	I am thinking about changing but haven't decided	I have plans to change but not yet working on it	I have started making changes to reach this goal	I have already reached this goal

I will get trained to give naloxone (or Narcan) in case someone I am with has an overdose.

I am not even thinking about this goal	I am thinking about changing but haven't decided	I have plans to change but not yet working on it	I have started making changes to reach this goal	I have already reached this goal

Risk behavior questionnaire

{Narrated text} In the next section I am going to ask you some questions about your drug use and other behaviors that might increase your risk of having hepatitis C. All your answers will be kept confidential and not shared with anyone outside the research team, so please be as honest as possible.

{Displayed text} Drug use questions = Confidential
How old were you the first time you injected any drug?
Enter number: _____

How many people do you know who shoot drugs?
Enter number: _____

In the past 6 months, how many of these people have you injected or shot drugs with? By this we mean you both shot up in the same location, whether or not you shared drugs or needles.
Enter number: _____

In the past, have you had sex with men, women, or both? This includes oral, vaginal or anal sex.
0 = Men only
1 = Women only
2 = Both men and women
3 = I have not had sex in the past

{Narrated text} For the next several questions, please indicate how long ago you did or experienced any of the following things. If a question refers to something you have not done in the past, you should select "never." When was the last time you shared needles, cottons, or cookers with someone else?

{Displayed text} Indicate how long ago you did or experienced any of the following things.
When was the last time you shared needles, cottons, or cookers with someone else?
0 = In the past 3 months
1 = Between 3 and 6 months ago
2 = Between 6 and 12 months ago
3 = More than 1 year ago
4 = Never

When was the last time you had sex with someone who shoots drugs?
0 = In the past 3 months
1 = Between 3 and 6 months ago
2 = Between 6 and 12 months ago
3 = More than 1 year ago
4 = Never

When was the last time you had sex with someone who you knew had hepatitis C?
0 = In the past 3 months
1 = Between 3 and 6 months ago
2 = Between 6 and 12 months ago
3 = More than 1 year ago
4 = Never

When was the last time you got a tattoo or body piercing from someone who was not a licensed professional?
0 = In the past 3 months
1 = Between 3 and 6 months ago
2 = Between 6 and 12 months ago
3 = More than 1 year ago
4 = Never

When was the last time you got someone else's blood on you during a fight, or any other significant blood exposure?
0 = In the past 3 months
1 = Between 3 and 6 months ago
2 = Between 6 and 12 months ago
3 = More than 1 year ago
4 = Never

When was the last time you were incarcerated in jail or prison?
0 = In the past 3 months
1 = Between 3 and 6 months ago
2 = Between 6 and 12 months ago
3 = More than 1 year ago
4 = Never

When was the last time you had 1 or more drinks containing alcohol?
0 = In the past 3 months
1 = Between 3 and 6 months ago
2 = Between 6 and 12 months ago
3 = More than 1 year ago
4 = Never

When was the last time you had an overdose from heroin, oxycodone, or some other narcotic drug?
0 = In the past 3 months
1 = Between 3 and 6 months ago
2 = Between 6 and 12 months ago
3 = More than 1 year ago
4 = Never

{Narrated text} Thank you. I'm next going to ask you about specific drugs you may have used recently. The questions in the next section will ask you to remember things about your drug use in the past 1 month.

{Displayed text} The questions in the next section will ask you to remember things about your drug use in the past 1 month.
In the past month, how often did you use heroin?
0 = Never
1 = About once a week or less
2 = More than once a week but not every day
3 = Every day

In the past month, how often did you use oxycodone or Oxycontin?
0 = Never
1 = About once a week or less
2 = More than once a week but not every day
3 = Every day

In the past month, how often did you use methamphetamine (crystal, or meth)?
0 = Never
1 = About once a week or less
2 = More than once a week but not every day
3 = Every day

In the past month, how often did you use cocaine or crack?
0 = Never
1 = About once a week or less
2 = More than once a week but not every day
3 = Every day

In the past month, how often did you drink alcohol
0 = Never
1 = About once a week or less
2 = More than once a week but not every day
3 = Every day

On the days you did drink, how many drinks would you have each day?
Enter number: _____

In the past month, have you used drugs more than you meant to?
0 = No
1 = Yes

In the past month, have you felt the need to cut down on your drug use?
0 = No
1 = Yes

[Skip if no heroin use in past month (SUBHER= 0)]
In the past month when you have used heroin, how often have you injected it?
0 = I never injected it
1 = About half of the time or less
2 = Most of the time
3 = Every time

[Skip if no oxy use in past month (SUBOXY= 0)]
In the past month when you have used Oxycontin or oxycodone, how often have you injected it?
0 = I never injected it
1 = About half of the time or less
2 = Most of the time
3 = Every time

[Skip if no meth use in past month (SUBCRYSTAL= 0)]

In the past month when you have used crystal or meth, how often have you injected it?
0 = I never injected it
1 = About half of the time or less
2 = Most of the time
3 = Every time

In the past month when you have used cocaine, how often have you injected it?
0 = I never injected it
1 = About half of the time or less
2 = Most of the time
3 = Every time

[Skip to SUBINJMAX if no opioid use in past month (SUBHER, SUBOXY, SUBMTHD all = 0)]

{Narrated text} We are next going to talk specifically about heroin, prescription narcotics such as oxycodone, and methadone. Drugs in this family are sometimes referred to as opioids. We are going to cover this important topic because opioids are the type of drug most likely to lead to an overdose, which can be deadly.

{Displayed text} **Opioids**:
Heroin
Oxycodone/Percocet
Hydrocodone/Vicodin
Methadone
Hydromorphone/Dilaudid

In the past month when you have used opioids, how often did you use when you were alone?
0 = Never
1 = A few times
2 = About half of the time
3 = Most of the time
4 = Every time

How often did you use opioids while you were also drinking alcohol?
0 = Never
1 = A few times
2 = About half of the time
3 = Most of the time
4 = Every time

How often did you have someone else inject opioids for you?
0 = Never
1 = A few times
2 = About half of the time
3 = Most of the time
4 = Every time

How often did you mix more than one type of drug in a syringe?
0 = Never
1 = A few times

2 = About half of the time
3 = Most of the time
4 = Every time

How often did you use opioids that you got from someone you didn't know?
0 = Never
1 = A few times
2 = About half of the time
3 = Most of the time
4 = Every time

How would you describe your current drug use (over the past month) compared to other times in your life when you were using?
0 = I am using more than I ever have
1 = I have always used this much
2 = I am using less than in past

Have you ever been given naloxone (also called Narcan) because of an overdose?
0 = No
1 = Yes
2 = I don't know

Have you ever been trained on how to give Narcan?
0 = No
1 = Yes

Have you ever given Narcan to someone else who had an overdose?
0 = No
1 = Yes

[Skip section if no opioid use in past month]

{Narrated text}The next 4 questions deal with how you think about preventing overdose. Listen to the following statements and choose the answer that indicates how much you agree or disagree:

{Displayed text} Choose the answer that indicates how much you agree or disagree."
"I am confident that if I really wanted to, I could prevent myself from having an overdose"

Strongly disagree	Disagree	Neutral	Agree	Strongly agree

"Most people who are important to me think that I should take steps to prevent overdose"

Strongly disagree	Disagree	Neutral	Agree	Strongly agree

"Most people who are similar to me are taking steps to prevent overdose"

Strongly disagree	Disagree	Neutral	Agree	Strongly agree

“Taking steps to prevent an overdose in the future is important to me”

Strongly disagree	Disagree	Neutral	Agree	Strongly agree

{Narrated text} Thanks. You are doing great. For the next section, think about all the times you shot up any drug <u>in the past month</u>.

{Displayed text} Think about all the times you shot up any drug <u>in the past 1 month</u>

When you shot up in the past month, how often did you use a new, unused needle?

0%	50%	100%
Never	About half of the time	Every single time

How often did you use a <u>needle</u> that had already been used by someone else?

0%	50%	100%
Never	About half of the time	Every single time

How often did you use a <u>needle</u> that you cleaned with bleach and water?

0%	50%	100%
Never	About half of the time	Every single time

How often did you use a cotton or filter that had already been used by someone else?

0%	50%	100%
Never	About half of the time	Every single time

How often did you use a <u>cooker</u> that had already been used by someone else?

0%	50%	100%
Never	About half of the time	Every single time

In the past month, how many different people have you shared needles with?
Enter number: _____

In the past month, how many people have you shared cottons or filters with?
Enter number: _____

In the past month, how many different people have you shared cookers with?
Enter number: _____

Think about the people you have shared needles or other works with in the past month. Do you know their hepatitis C status?

0 = I know that none of them has hepatitis C
1 = I know that at least one person has hepatitis C
2 = Some of them may have had hepatitis C, but I don't know for sure

{Narrated text} The next questions ask what you think about changing the way you shoot up to keep yourself or the people you shoot with from getting hepatitis C, HIV, and other health problems. Listen to the following statements and choose the answer that indicates how much you agree or disagree:

{Displayed text} Listen to the following statements and choose the answer that indicates how much you agree or disagree:

"I am confident that if I really wanted to, I could use clean needles, cottons, and cookers every single time I shoot up."

Strongly disagree	Disagree	Neutral	Agree	Strongly agree

"Most people who are important to me think that I should use clean needles, cottons, and cookers every time I shoot up"

Strongly disagree	Disagree	Neutral	Agree	Strongly agree

"Most people who are similar to me use clean needles, cottons, and cookers every time they shoot up"

Strongly disagree	Disagree	Neutral	Agree	Strongly agree

"Using clean needles, cottons, and cookers every time is important to me."

Strongly disagree	Disagree	Neutral	Agree	Strongly agree

HCV Testing
(skip section if known HCV positive)

Before today, how many times in your life have you been tested for hepatitis C? If you were tested today for the first time, enter zero.
Enter number: _____

What are some of the reasons you have not been tested for hepatitis C regularly in the past? Check all that apply?
0 = I never thought about having hepatitis C / getting tested never crossed my mind
1 = I didn't think there was a chance I had hepatitis C
2 = My doctor never suggested it or asked if I wanted to get tested
3 = I didn't want to know if I had it
4 = I didn't have time to get tested
5 = I thought people would judge me if I asked to get tested
6 = I don't like getting my blood drawn
7 = I didn't know where I could go to get tested

8 = I was afraid about how people would treat me if they knew I had hepatitis C
9 = It wouldn't make a difference whether I have hepatitis C or not

If agreed to HCV test today:
How likely are you to get tested for hepatitis C again six months from now?

Very unlikely	Somewhat unlikely	Neither unlikely nor likely	Somewhat likely	Very likely

If participant did not agree to HCV test today:
How likely are you to get tested for hepatitis C in the next six months?

Very unlikely	Somewhat unlikely	Neither unlikely nor likely	Somewhat likely	Very likely

{Listen to the following statements and choose the answer that indicates how much you agree or disagree:}

"I am confident that if I really wanted to, I could get tested for hepatitis C every six months, for as long as I am shooting drugs."

Strongly disagree	Disagree	Neutral	Agree	Strongly agree

"Most people who are important to me think I should get tested for hepatitis C."

Strongly disagree	Disagree	Neutral	Agree	Strongly agree

"Most people who are similar to me have been tested for hepatitis C."

Strongly disagree	Disagree	Neutral	Agree	Strongly agree

"Getting tested for hepatitis C is important to me."

Strongly disagree	Disagree	Neutral	Agree	Strongly agree

Mental health screen

In the last 2 weeks, how often have you been bothered by the following problems?
1. less interest or pleasure in doing things you used to enjoy
0= Not at all
1= Several days
2= More than half the days
3= Nearly everyday

In the last 2 weeks, how often have you been bothered by the following problems?
2. Feeling down, depressed, or hopeless
0= Not at all
1= Several days
2= More than half the days
3= Nearly everyday

Do you often worry or feel nervous?
0 = No
1 = Yes

Are you often fearful of interacting with other people?
0 = No
1 = Yes

Do you ever feel jittery, short of breath, or like your heart is racing?
0 = No
1 = Yes

Do you ever feel as if you might lose control or fear that you may be "losing" it?
0 = No
1 = Yes

In your life, have you ever had any experience that was so upsetting, frightening or horrible that, <u>in the past month</u>, you:
Had nightmares about it or think about it when you do not want to?
0 = No
1 = Yes

Try hard not to think about it or go out of your way to avoid situations that remind you of it?
0 = No
1 = Yes

Are constantly on guard, watchful or easily startled?
0 = No
1 = Yes

Feel numb or detached from others, activities or your surroundings?
0 = No
1 = Yes

Health care access and utilization

{Narrated text} You are just about done. The last few questions ask about your current living and financial situation. Some of these things may help determine whether you qualify for free or low-cost health insurance.

Who pays for your health care?
0 = I don't have any health insurance
1 = Public assistance (BadgerCare, Medicaid, Medicare)
2 = Employer
3 = Military/VA
4 = Parents
5 = School
6 = Ryan White / ADAP (HIV drug assistance)
7 = I pay for my own health insurance
8 = Other

Do you have a doctor or other provider that you see for routine check-ups or physicals?
0 = No
1 = Yes

Have you ever received a vaccine for hepatitis A?
0 = No
1 = Yes
2 = I don’t know

Have you ever received a vaccine for hepatitis B?
0 = No
1 = Yes
2 = I don’t know

If participant is HCV-positive:
Have you ever talked with a doctor who is a liver specialist about getting treatment for hepatitis C?
0 = No
1 = Yes

Skip unless the participant does not have health insurance:
During the next year, how likely is it that you will get health insurance?
0 = Very unlikely
1 = Somewhat unlikely
2 = Unsure
3 = Somewhat likely
4 = Very likely

Do you have a job?
0 = No
1 = Yes, part time
2 = Yes, full time

Do you have any children who live with you?

0 = No
1 = Yes

How much legal income did you have in the past year?
0 = none
1 = Less than $11,500
2 = $11,500 to $15,000
3 = $15,000 to $20,000
4 = $20,000 to $30,000
5 = $30,000 to $40,000
6 = More than $40,000

Did you file a tax return last year?
0 = No
1 = Yes

Have you been homeless at all during the past year?
0 = No
1 = Yes

[Randomization occurs]

End for control group:
{Narrated text} We've reached the end of today's program. Thank you for taking the time to participate today. Please give the computer back to the prevention specialist. Remember to come back for the second part of the study 3 months from now. Someone from the study will contact you to remind you when it is time to come back. Until then, take care and stay safe!}

End for intervention group:
{Narrated text} Thanks for completing the survey. These issues can be hard to talk about, so I really appreciate your honesty.

You've been selected to complete the second part of this computer program, which was designed to help people think about how to be safer and healthier when it comes to injecting drugs.
