# Supplementary material for: Computerized Tailored Interventions to Enhance Prevention and Screening for Hepatitis C Virus Among People Who Inject Drugs: Protocol for a Randomized Pilot Study
Source: JMIR Res Protoc. 2016 Jan 22;5(1):e15. doi: 10.2196/resprot.4830 (PMC4744331; doi:10.2196/resprot.4830)
Supplement: Supplementary file 2 [file resprot_v5i1e15_app2.pdf]

Appendix 2. Sample content screens from Hep-Net intervention
(Hypothetical participant reporting daily heroin use, pre-contemplation stage for completing naloxone training)

Computer Screen Displays | Narrated text

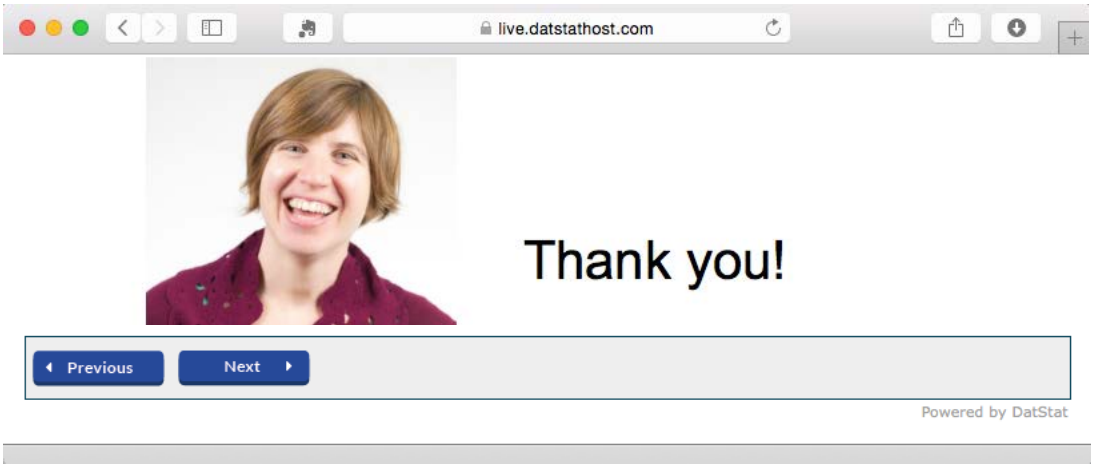

"Thanks for completing the survey. These issues can be hard to talk about, so I really appreciate your honesty.

You've been selected to complete the second part of this computer program, which was designed to help people think about how to be safer and healthier when it comes to injecting drugs."

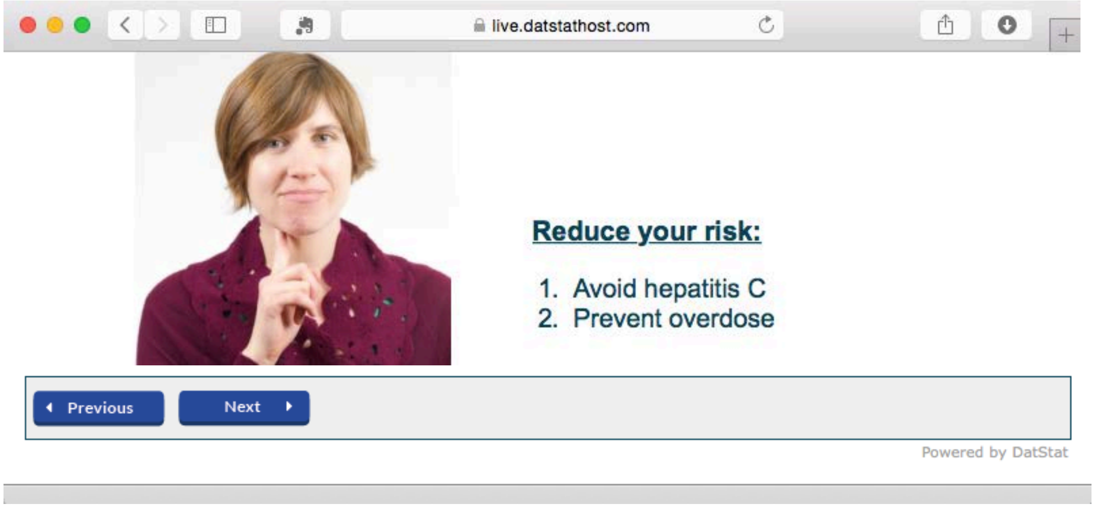

Risk Synthesis

"Most people who use drugs care about their health, even though they sometimes use in unhealthy ways. Injecting drugs can lead to many different kinds of health problems. Today we are going to focus on 2 of these: hepatitis C and other infections, and opioid overdose."

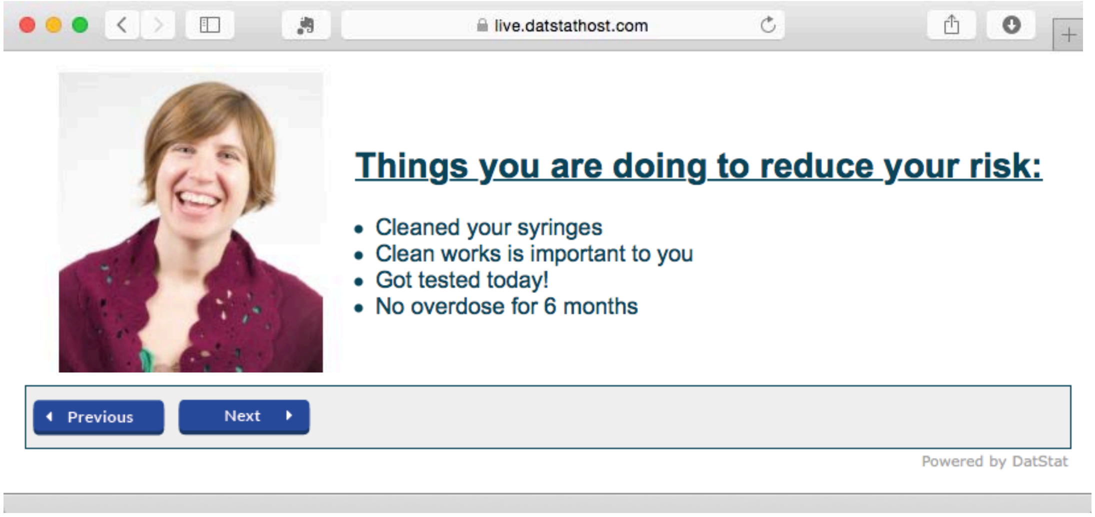

"Based on the responses you gave during the survey, I see that you are already doing a number of things that can lower your risk of infection or overdose. Let's review some of the healthy behaviors you told us about."

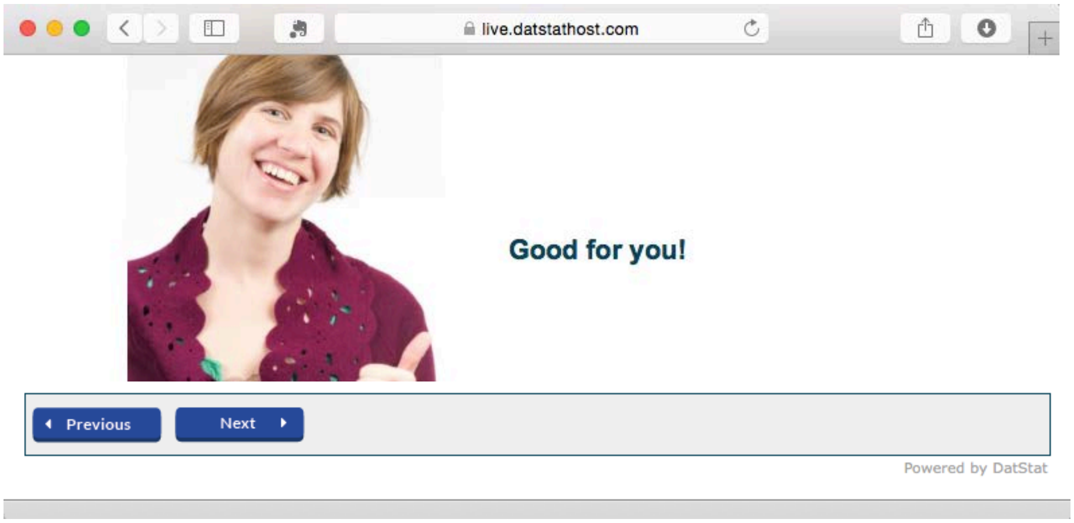

"It is great to know that you have been doing these things to keep yourself healthy. It shows that you care about your health and are willing to take steps to avoid some of the harmful consequences of using drugs. Injecting drugs can never be completely safe, however. Now we are going to take a look at the things you told us about that can increase your risk."

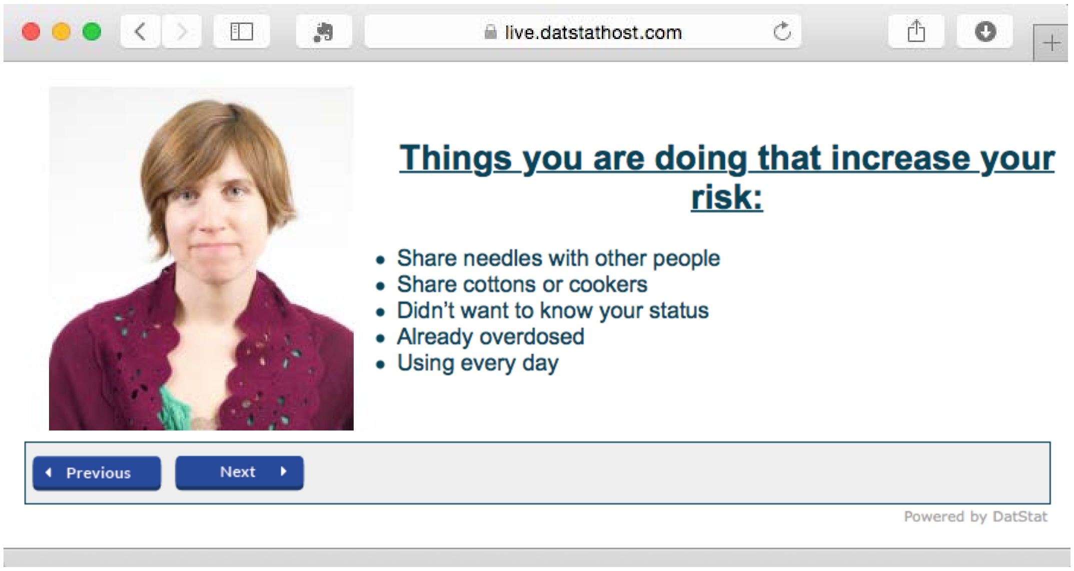

"Here are some of the things you told us about that could lead to health problems in the future."

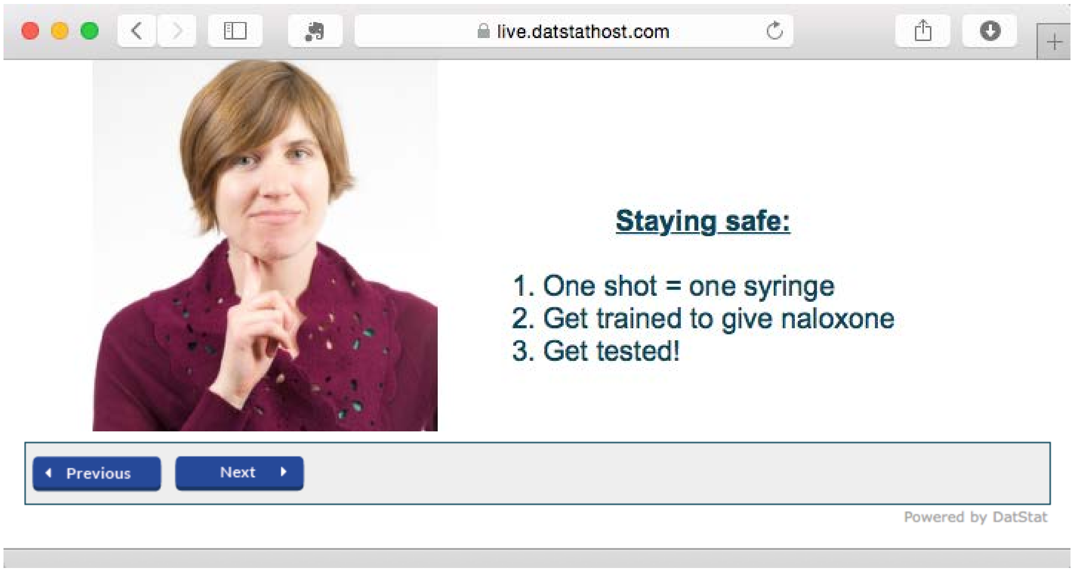

"Based on what you told us in the survey, there are definitely some things you can do to lower your risk of having health problems from drug use. If you are not ready to quit using drugs, remember these 3 things to stay safer:
1. Use a new needle every time and don't share cottons or cookers
2. Get trained to give Naloxone in case someone you are with overdoses
3. Get tested for hepatitis C and HIV, and encourage your friends to get tested too.

Next, I'm going to guide you through the process of picking an area to work on that could lower your risk of health problems."

Selection of behavioral goal

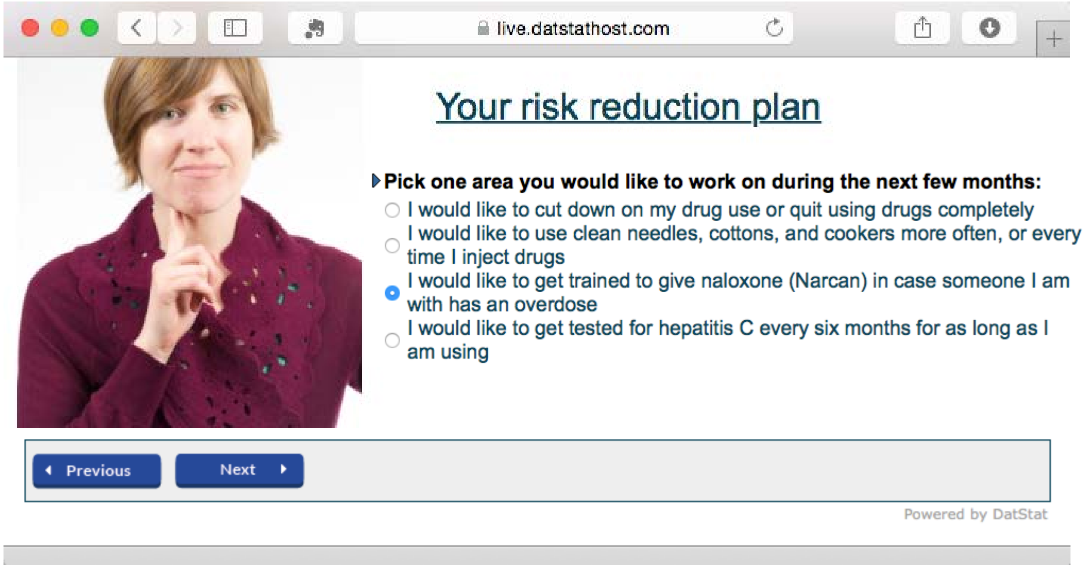

"There are many steps you can take that can make a big difference in keeping you, your friends and partners safer and healthier when using drugs. I'm now going to help you create your own plan for reducing your risk in the future. To start, please pick one of the following areas that you think you could work on during the next few months."

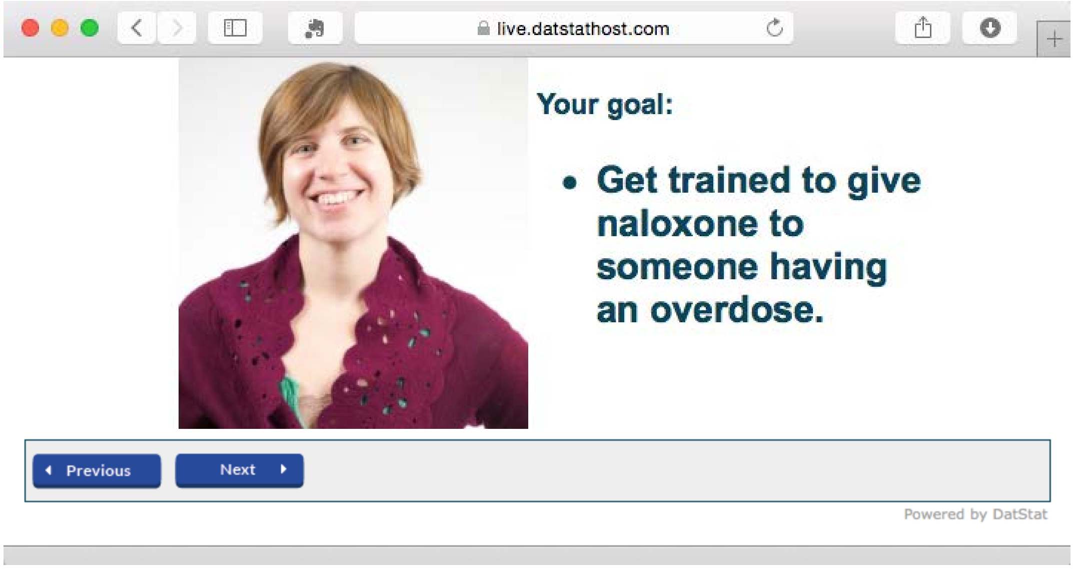

"You picked "I would like to get trained to give naloxone (Narcan) in case someone I am with has an overdose." Great choice! Let's see what we can do to help you as you work on this goal."

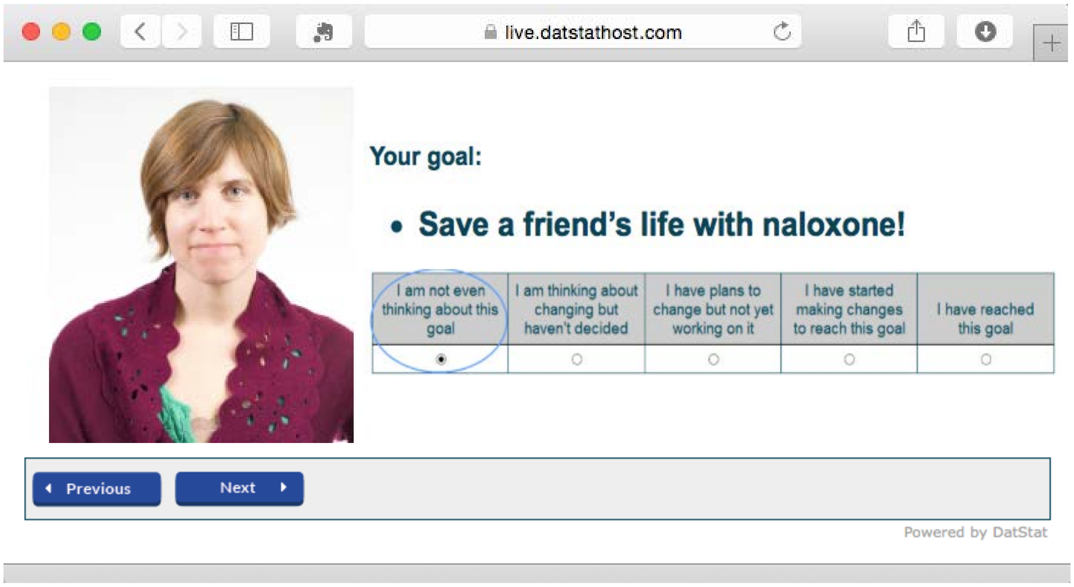

"In the survey you just took, you indicated that haven't thought about getting trained to give naloxone to someone who overdoses. You are not alone, many people have either never heard of naloxone or just never considered giving naloxone to someone who overdoses. Since you chose this area to work on during the next few months, it sounds like you are starting to think about naloxone training yourself. That's great!"

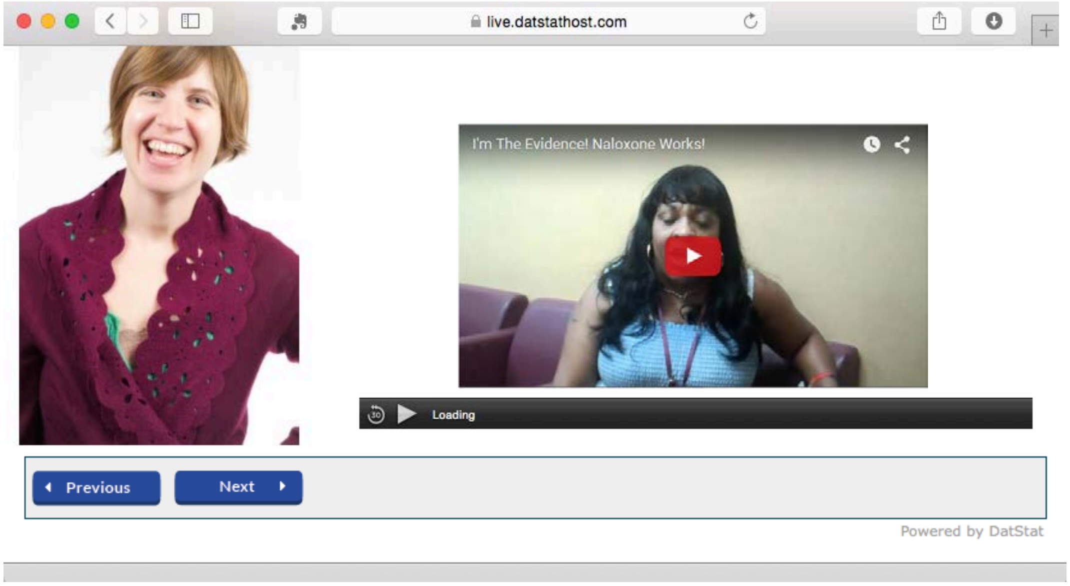

"To hear more, click on the graphic below to play a short video about people who have been rescued from an overdose with naloxone. You'll learn that you too can save a friend or loved one from dying from an opioid overdose."

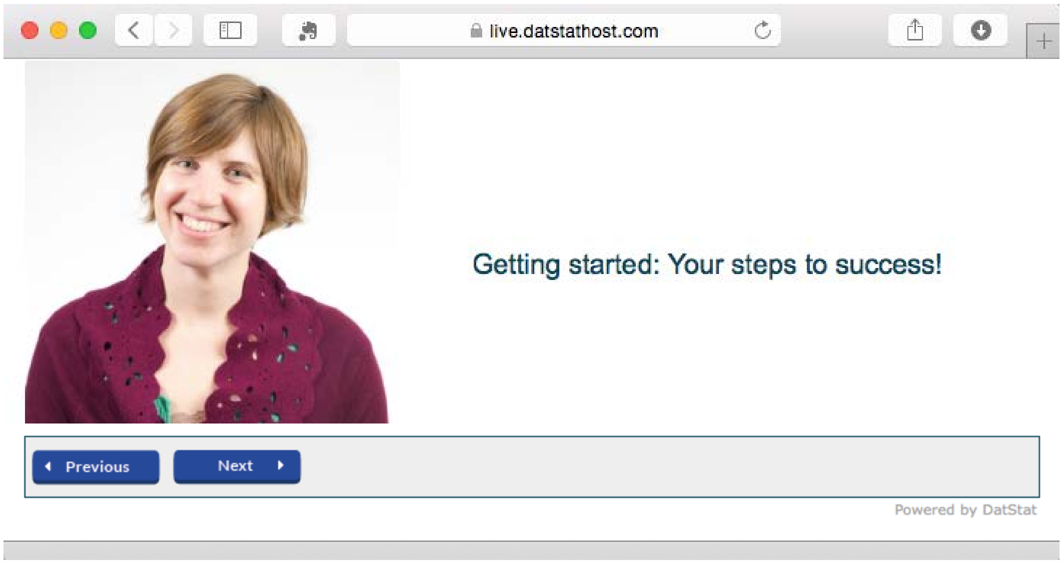

Individualized risk reduction exercise

"You're doing great! While it is great to have a goal to improve your health, achieving a goal can be hard. A common challenge is when someone knows the change he or she wants to make, but doesn't know how to get started. Next we are going to go over a list of steps that you could take to get you closer to your meeting your goal. After we go through these, I'd like you to pick several of them, and commit to trying them during the next few months."

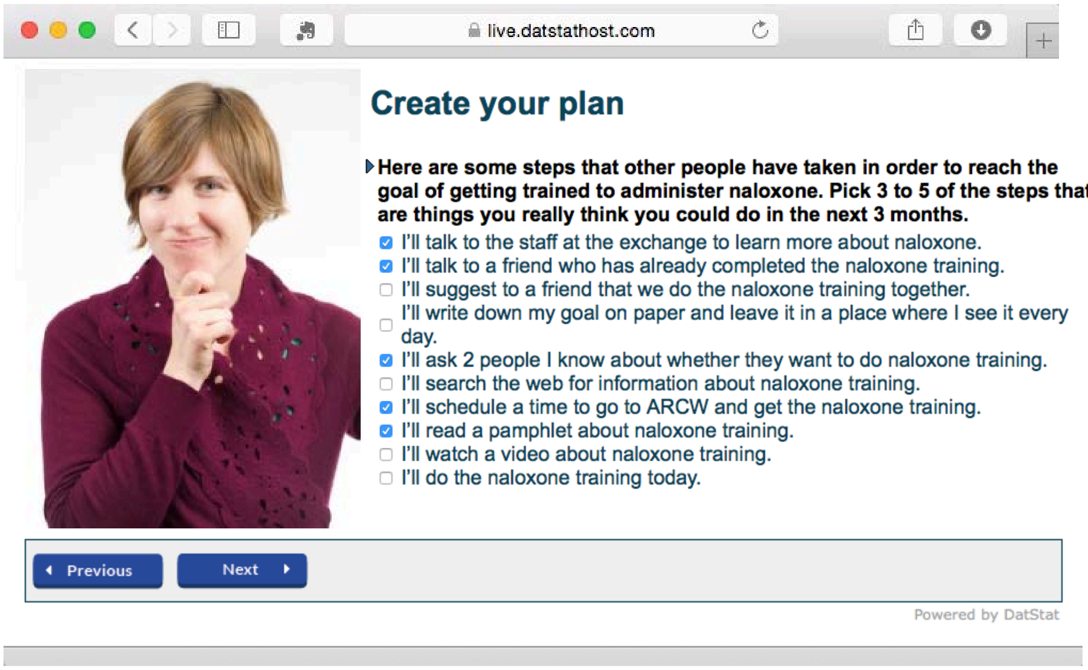

"Here are some steps that other people have taken in order to reach the goal of getting trained to administer naloxone. Pick 3 to 5 of the steps that are things you really think you could do in the next 3 months."

live.datstathost.com

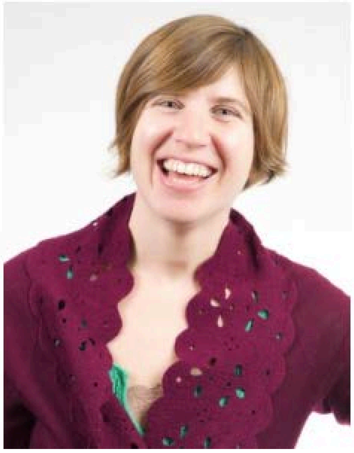

Review your action plan:

- I'll talk to the staff at the exchange to learn more about naloxone
- I'll talk to a friend who has already completed the naloxone training
- I'll ask 2 people I know about whether they want to do naloxone training.
- I'll schedule a time to go to ARCW and get the naloxone training
- I'll read a pamphlet about naloxone training

Previous Next

Powered by DatStat

"Great. Let's review the steps you picked.

Let's make these steps your personal "action plan" for the next three months as you work toward you goal. When you come back for the second half of this study in three months, I will ask you how it went! Good luck! I have just a few more questions before we are done for today."

live.datstathost.com

When making a plan to change something in our lives, it's really helpful to commit to a "start date."
When will you start?

- ○ Today
- ● Tomorrow
- ○ Later this week
- ○ This weekend
- ○ The beginning of next month
- ○ Some other time (specify:) ______
- ○ I choose not to answer

Previous Next

Powered by DatStat

live.datstathost.com

Many people find it helps to get support to carry out their plan. Who might support you or help you in doing your plan?

- ☑ Friends. List first initial of friend(s): M
- ☐ Parents, brother or sister
- ☐ Doctor
- ☐ Clinic staff
- ☑ My boyfriend/girlfriend/spouse/partner
- ☐ Hotline or chatline
- ☑ Exchange staff
- ☐ Case manager/social worker
- ☐ I choose not to answer

Previous Next

Powered by DatStat

live.datstathost.com

Is there anything you can think of that will get in the way of you doing your plan?
Choose all that apply:

- [x] My friends/partners won't like it
- [ ] I won't have time
- [ ] I might forget
- [ ] I'm not really sure I want to do it
- [ ] It might be uncomfortable for me (or it might cause me emotional or physical pain that I don't feel I can handle on my own)
- [x] I need someone to talk with
- [x] I might feel too sick
- [ ] I might not be able to afford it
- [ ] I'm worried about my boyfriend/girlfriend/spouse/partner's reaction
- [ ] My friends/partners won't cooperate
- [ ] I feel I need more skills
- [ ] I am scared I might fail
- [ ] I don't think anything will get in the way of doing my plan
- [ ] Other
- [ ] I choose not to answer

Previous Next

Powered by DatStat

live.datstathost.com

What would help you get over this barrier?

- ( ) Get more information
- ( ) Keep my plan with me (a paper print out that you can get from exchange staff)
- (•) Get support
- ( ) Talk to someone at the clinic or exchange
- ( ) Other
- ( ) I choose not to answer

Previous Next

Powered by DatStat

live.datstathost.com

Does this plan sound like something you can do?

- ( ) Definitely yes
- (•) I think so
- ( ) I'm not sure
- ( ) Maybe not
- ( ) Definitely no
- ( ) I choose not to answer

Previous Next

Powered by DatStat

live.datstathost.com

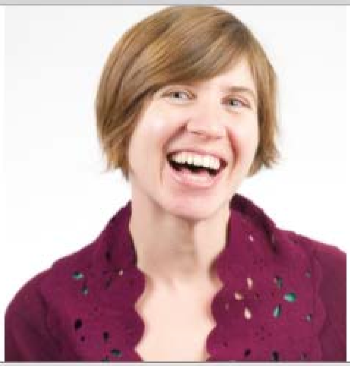

Congratulations & Good Luck!

Previous | Next

Powered by DatStat

"Congratulations! Great work - you've just taken a huge step toward a healthier life. Thank you for taking the time to participate today. I hope you found it useful. Good luck working on your goal. I look forward to hearing how it went when you come back for the second part of the study in 3 months. Until then, take care and stay safe!"

live.datstathost.com

Hep-Net Wisconsin
Individualized risk reduction plan for TT.
10/19/2015, 8:54 PM

Your Prevention Goal:

I would like to get trained to give naloxone (Narcan) in case someone I am with has an overdose

Your Risk Reduction Action Plan:

- I'll talk to the staff at the exchange to learn more about naloxone
- I'll talk to a friend who has already completed the naloxone training
- I'll ask 2 people I know about whether they want to do naloxone training.
- I'll schedule a time to go to ARCW and get the naloxone training
- I'll read a pamphlet about naloxone training

Your Start Date:

Tomorrow

Your Source of Support:

Friends. List first initial of friend(s):, My boyfriend/girlfriend/spouse/partner, Exchange staff

Good luck putting your plan into action!

Don't forget your next study appointment in 3 months.

Next Study Appointment Due Date: 1/17/2016
Questions? Call

Print this summary

Previous | Next

Powered by DatStat
