# Supplementary material for: Computerized Tailored Interventions to Enhance Prevention and Screening for Hepatitis C Virus Among People Who Inject Drugs: Protocol for a Randomized Pilot Study
Source: JMIR Res Protoc. 2016 Jan 22;5(1):e15. doi: 10.2196/resprot.4830 (PMC4744331; doi:10.2196/resprot.4830)
Supplement: Supplementary file 3 [file resprot_v5i1e15_app3.pdf]

Appendix 3. Follow-up Assessment

*Note: Risk behavior questions delivered during the baseline questionnaire are repeated during the follow-up study encounter.

{Narrated text} There are many steps you can take that can make a big difference in keeping you, your friends and partners safer and healthier when using drugs. In the past 3 months, did you consider taking any steps to meet the following goals?

Cut down on my drug use or quit using drugs completely.

0 = No, not at all
1 = I thought about this goal but didn't take action
2 = I tried some things but have not met this goal yet.
3 = Yes, I met this goal

Use clean needles, cottons, and cookers more often, or every time I inject drugs.
0 = No, not at all
1 = I thought about this goal but didn't take action
2 = I tried some things but have not met this goal yet
3 = Yes, I met this goal

Get trained to give naloxone (Narcan) in case someone I am with has an overdose (Not displayed if already received naloxone training prior to baseline visit)
0 = No, not at all
1 = I thought about this goal but didn't take action
2 = I tried some things but have not met this goal yet
3 = Yes, I met this goal

Get tested for hepatitis C every six months for as long as I am using (Not displayed if already HCV positive)
0 = No, not at all
1 = I thought about this goal but didn't take action
2 = I tried some things but have not met this goal yet
3 = Yes, I met this goal

Follow-Up questions <u>for HCV-positive participants ONLY</u>

In the past 6 months, did you find out for the first time that you were hep C positive?
0=No
1=Yes
2= I don’t know

Did you see any medical provider to talk about your hepatitis C diagnosis?
0=No
1=Yes

Why did you not see a medical provider ?
0=I couldn’t afford it
1=I don’t know of any providers I could see in my area
2=I didn’t have time
3=I didn’t think it was important
4=I was afraid
5=Other____________________

What was the name of the clinic or hospital where you saw a provider to discuss hepatitis C?
Enter text:_________

Did you see a liver specialist or infectious disease specialist to discuss getting treated for hepatitis C?
0=No
1=Yes

Did you start taking any medications to treat hepatitis C?
0=No
1=Yes

Have any of these things changed <u>because</u> you learned about your hepatitis C status
0= How often I inject drugs
1= How often I share works when I inject
2= How often I talk to people about hepatitis C
3= How often I use the needle exchange

Follow-up questions for <u>intervention group ONLY</u>

{Narrated text} You're doing great, we're just about finished. Listen to the following statement and choose the answer that indicates how much you agree or disagree.

"I found the Hep-Net program easy to use."

Strongly disagree	Disagree	Neutral	Agree	Strongly agree

"I trust that the information I entered into the Hep-Net program will be kept confidential."

Strongly disagree	Disagree	Neutral	Agree	Strongly agree

"I prefer answering questions about my drug use using a computer program rather than through an in-person interview."

Strongly disagree	Disagree	Neutral	Agree	Strongly agree

"The Hep-Net program gave me information that was specific to my needs."

Strongly disagree	Disagree	Neutral	Agree	Strongly agree

"This program changed the way I think about my drug use."

Strongly disagree	Disagree	Neutral	Agree	Strongly agree

"I will be safer when I shoot up as a result of this program."

Strongly disagree	Disagree	Neutral	Agree	Strongly agree

"I felt comfortable asking people I know to sign up for this study."

Strongly disagree	Disagree	Neutral	Agree	Strongly agree
